# Job satisfaction and intent to stay in neonatal nursing in England and Wales: a study protocol

**DOI:** 10.1186/s12913-024-11379-0

**Published:** 2024-08-08

**Authors:** Kathy Chant, Jos M. Latour, Nicola Booth, Lisa Viola, Kelly Crofts, Yoko Nishimura, Katie Gallagher

**Affiliations:** 1 Division of Academic Neonatology, Institute for Women’s Health, University College London, London, UK; 2 School of Nursing and Midwifery, Faculty of Health, University of Plymouth, Plymouth, UK; 3 Curtin School of Nursing, Curtin University, Perth, Australia; 4 Neonatal Intensive Care Unit, Manchester University NHS Foundation Trust, Manchester, UK; 5 Neonatal Intensive Care Unit, Milton Keynes University Hospital NHS Foundation Trust, Milton Keynes, UK; 6 Neonatal Intensive Care Unit, University Hospital of Wales, Cardiff, UK; 7 Neonatal Intensive Care Unit, University College London Hospitals NHS Foundation Trust, London, UK

**Keywords:** Nursing retention, Neonatal nursing, Job satisfaction, Burnout, Intent to stay

## Abstract

**Background:**

Nursing shortages are an ongoing concern for neonatal units, with many struggling to meet recommended nurse to patient ratios. Workforce data underlines the high proportion of neonatal nurses nearing retirement and a reduced number of nurses joining the profession. In order to recommend strategies to increase recruitment and retention to neonatal nursing, we need to understand the current challenges nurses are facing within the profession. The aim of this study is to investigate current job satisfaction, burnout, and intent to stay in neonatal nursing in England and Wales.

**Methods:**

This study has two parts: (1) a systematic review exploring job satisfaction, burnout and intent to stay in neonatal nursing, and any previous interventions undertaken to enhance nurse retention, (2) an online survey of neonatal nurses in England and Wales exploring job satisfaction, burnout and intent to stay in neonatal nursing. We will measure job satisfaction using the McCloskey Mueller Satisfaction Scale (MMSS), burnout using the Copenhagen Burnout Inventory (CBI) and the Nurse Retention Index (NRI) will be used to measure intent to stay. All nurses working in neonatal units in England and Wales will be eligible to participate in the nursing survey.

**Discussion:**

Retention of neonatal nurses is a significant issue affecting neonatal units across England and Wales, which can impact the delivery of safe patient care. Exploring job satisfaction and intent to stay will enable the understanding of challenges being faced and how best to support neonatal nurses. Identifying localised initiatives for the geographical areas most at risk of nurses leaving would help to improve nurse retention.

## Background

A combination of low recruitment and high turnover rates of registered nurses has contributed to a global shortage of nurses [1]. Combined with the number of nurses nearing retirement, the World Health Organisation (WHO) estimates a global shortfall of around 9 million by 2030 [2]. Across the UK, there are an estimated 50,000 nursing vacancies, with 46,828 in England [3], and 1,719 in Wales [4]. At a time when healthcare demand has risen owing to an aging population, and an increase in patients with complex care needs, nursing retention is of critical importance. Evidence exploring reasons for leaving nursing is limited and complex, however factors such as retirement, work life balance, and health issues have been reported [5]. A recent national survey of nurses considering leaving revealed nurses felt undervalued, understaffed, and exhausted [6].

The provision of safe care is of importance within neonatal nursing. Critically ill infants cared for in the neonatal intensive care unit (NICU) are among the most vulnerable patient group within a hospital, requiring specially trained nurses to care for them. There are currently around 5000 neonatal nurses working across almost 200 neonatal units in the UK [7], however due to national staff shortages, only 71% of units meet nurse to patient ratios as recommended by the British Association of Perinatal Medicine [8]. Research has explored the implications of these workforce challenges, with increased neonatal nursing workload associated with greater opportunities for missed nursing care, increased risk of infant health care associated infections, and increased patient safety concerns [9–11]. Nursing workload has also been associated with job satisfaction and intent to stay in the profession, with high patient to nurse ratios and missed nursing care associated with greater job dissatisfaction and increased odds of nurses intending to leave [12, 13]. Workplace stress, low morale, and an over-reliance on nurses to fill gaps in rotas as agency or bank shifts, or on the goodwill of nurses to keep services running [14] continue at a time when nurses are still recovering from the extra strain of the COVID-19 pandemic which saw many neonatal nurses re-deployed to adult intensive care units due to their critical care skill set.

Neonatal nursing workforce challenges are exacerbated by the complex and emotionally challenging environment of the neonatal unit [15]. As part of a larger team neonatal nurses are responsible for caring for extremely preterm and/or critically unwell infants, potentially having frequent conversations with parents around life and death decisions [16]. Advancing technology has resulted in the ability to provide treatment to babies born at the edge of viability from 22 weeks gestation onwards, creating ethically challenging scenarios which often result in differing attitudes towards how treatment should be provided to families [17, 18]. In a qualitative study of neonatal nurses in Sweden, concerns for patient safety when workload was heavy were expressed [19]. Nurses reported feelings of guilt and inadequacy at being unable to provide the attention and support they felt families required. This increased empathetic engagement can result in increased rates of secondary post-traumatic stress and burnout in comparison to adult critical care nurses, and increases with age and experience [20]. Moral distress has been linked to staff burnout, which in turn impacts staff turnover.

It is therefore imperative to explore current job satisfaction and intent to stay in neonatal nursing to identify the risk of increased attrition in nursing numbers, and the potential future impact upon neonatal services. Interventions to increase nurse retention have largely focussed on adult nurses or early career nurses and range from team building [21], to professional identity development programmes [22] and mentorship programmes [23], each with varying success. To our knowledge there have been few interventions to improve retention in neonatal nursing and few studies have focused on job satisfaction, burnout, and intent to stay amongst neonatal nurses, despite recommendations to improve recruitment and retention [24]. This study will therefore investigate current job satisfaction, burnout, and intent to stay in neonatal nursing. It will also explore previously trialled interventions to increase nurse retention, and obtain neonatal nurses’ opinions on these interventions. Results from the study will inform future policy for increasing retention rates amongst neonatal nurses.

## Methods

### Aim

The aim of this study is to explore job satisfaction, burnout, and intent to stay in neonatal nursing in England and Wales.

### Design

There are two strands to this study:

1. A systematic review of current literature

A systematic review of the literature will be undertaken to explore job satisfaction, burnout, and intent to stay in neonatal nursing. We will also identify the impact of any initiatives to improve retention in nursing. The review will be registered in the International Prospective Register of Systematic Reviews (PROSPERO), using PRISMA guidelines to assist with the development of the protocol. The search strategy for the review will be developed using defined MeSH descriptor headings and undertaken in all relevant databases, with no date or language limitations. Papers not written in English will be translated either through the hosting search database such as EBSCOHost or following upload to Google translate. All studies meeting the inclusion criteria will be reviewed by 2 reviewers independently (KC, YN) and any discrepancies discussed with a third (KG). Data from eligible studies will be extracted into a pre-designed outcome proforma (including author, year, country of study, study design, study intervention, characteristics of study intervention, participants and sample size, and outcome measures). The methodological quality of the studies will be assessed using the relevant criteria (Critical Appraisal Skills Programme and the Joanna Briggs Institute Critical Appraisal tool).

2. Neonatal nursing survey

Validated scales will be used to explore job satisfaction, burnout and intent to stay. Job satisfaction will be measured using a the 31 item McCloskey Mueller Satisfaction Scale (MMSS) [25]. The survey contains 5 point Likert scale questions ranging from very dissatisfied (1) to very satisfied (5) measuring 8 domains for nurses job satisfaction: satisfaction with extrinsic rewards, scheduling, family/work balance, co-workers, interaction, professional opportunities, praise/recognition, and control/responsibility. Intent to stay in nursing will be measured using the Nursing Retention Index (NRI) [26]. The survey is comprised of 6 items measured using an 8-point Likert scale from 1 (definitely false) to 8 (definitely true). Permission has been sought from the author to contextualise the questions to neonatal nursing. For example, item 4 ‘I expect to keep working as a nurse’ will be changed to ‘I expect to keep working as a neonatal nurse’. Neonatal nurses may have received paediatric, adult or midwifery training; we would like to measure the responses for nurses who intend to leave the area of neonatal care rather than the nursing or midwifery profession. Burnout will be measured using the Copenhagen Burnout Inventory (CBI) which is a 19 item survey measured using a five point Likert scale [27]. The CBI measures three different areas of burnout: personal (six items), work-related (seven items) and client-related (six items), and has been used previously to explore burnout in the nursing population.

We will collect demographic information including gender, neonatal unit, length of time since qualification, highest qualification, number of working hours per week, and banding level. Based on the results of the systematic review, further questions will be developed to explore nurses’ attitudes towards previously trialled interventions to improve nursing retention. This will likely be comprised of 5-point Likert scale and open-ended questions.

### Participants

All neonatal nurses, nurse associates, nursery nurses and healthcare assistants working on neonatal units in England and Wales will be eligible to take part in the study. Student nurses, nurses working outside of England and Wales, and nurses working outside of a neonatal unit will not be included in the study. There are 5000 neonatal nurses working in the UK, however it is unclear how many of these nurses work in England and Wales. We aim to recruit over 1000 neonatal nurses to the study.

### Study procedure

The systematic review will run alongside the application for ethical approval. Neonatal nurses will be recruited through their hospital; neonatal units agreeing to advertise the study will send an email to all neonatal nurses and place a poster in nursing staff break rooms with a link or QR code signposting to the study information. Recruitment will be enhanced through advertisement in email, newsletters, and the social media platform of relevant professional bodies including the Neonatal Nurses Association (NNA). We are also collaborating with the lead neonatal nurse in the National Health Service England (NHSE) and neonatal nursing research and practice champions from around the country, who will further promote the study.

The survey will be hosted by REDCap, a secure platform for managing online surveys. Written consent will be requested at the start of the online survey and required to access the survey questions. The survey will take approximately 15 minutes to complete. Any identifying information provided will be removed prior to data analysis to ensure anonymity. The study will be open to responses for 7 months between March and September 2024.

The study has received ethical approval by the Health Research Authority (HRA) and the Health and Care Research Wales ((HCRW), HRA ID: 23/HRA/4690). The study has also been registered under the National Institute for Health Research (NIHR) Portfolio which provides support for funded research studies conducted in hospitals in England and Wales.

### Data analysis

Data from the surveys will be imported into SPSS for data analysis. Descriptive statistics will be performed per questionnaire, and mean scores will be measured per item/subscale. Internal reliability will be measured using Cronbach’s alpha. Correlations between job satisfaction and burnout with intent to stay will be assessed using Pearson’s correlation. Multivariate regression will be used to examine the relationship between subscales of job satisfaction and burnout with intent to stay to identify the greatest predictors. Open ended questions will be analysed using content or thematic analysis, depending on the amount of text involved, looking either for repeated words, phrases or themes between the text [28]. Results will be reported using the CROSS reporting guidelines [29].

## Discussion

Neonatal nursing staff retention is currently a significant issue facing most neonatal units throughout England and Wales, with potential significant impacts upon the delivery of safe and effective care of babies and their families admitted for neonatal care. This study will identify the experience of neonatal nurses across England and Wales, through measures of neonatal nursing job satisfaction, burnout and intent to stay in neonatal nursing. This will enable us to identify geographical areas, or neonatal networks, at high-risk for staff losses, and the reasons behind this. Exploring the attitudes and experiences of neonatal nurses towards previously trialled initiatives to improve nursing job satisfaction, burnout and retention, will allow us to determine what initiatives could potentially improve nursing job satisfaction and retention in the future. This could lead to future research to determine the feasibility of a specific intervention to enhance neonatal nursing retention. The approach of this study could also be used as a framework for other specialist nursing areas to similarly explore workforce and retention in their own area.

## Data Availability

No datasets were generated or analysed during the current study.

## Abbreviations

CBI: Copenhagen Burnout Inventory
DHSC: Department of Health and Social Care
GIRFT: Getting it Right First Time
HCRW: Health and Care Research Wales
HRA: Health Research Authority
MMSS: McCloskey Mueller Satisfaction Scale
NHSE: National Health Service England
NIHR: National Institute for Health Research
NNA: Neonatal Nurses Association
NRI: Nurse Retention Index
PROSPERO: International Prospective Register of Systematic Reviews
RCN: Royal College of Nursing
RCPCH: Royal College of Paediatrics and Child Health
WHO: World Health Organisation
